# Preconception Immunoglobulins and Complements as Potential Biomarkers in Unexplained Female Infertility in Saudi Arabia

**DOI:** 10.7759/cureus.48322

**Published:** 2023-11-05

**Authors:** Emad A Koshak, Hosam Aljohani, Ali Atwah, Rajeh Aljedani, Yasser Aljaied, Mahmoud A Gaddoury

**Affiliations:** 1 Internal Medicine, King Abdulaziz University Hospital, Jeddah, SAU; 2 Pediatrics, King Abdulaziz University Hospital, Jeddah, SAU; 3 Medicine, King Abdulaziz University Hospital, Jeddah, SAU; 4 Family Medicine, King Abdulaziz University Hospital, Jeddah, SAU; 5 Community Medicine, Faculty of Medicine, King Abdulaziz University, Jeddah, SAU

**Keywords:** immunoglobulin m, immunoglobulin g, immunoglobulin a, complement c4, complement c3, unexplained female infertility

## Abstract

Background: Immunological abnormalities are currently under investigation to potentially unravel the etiology of frustrating cases of unexplained female infertility (UFI).

Objectives: To explore the prevalence of immunological abnormalities in the levels of total immunoglobulins and complements in the cases of UFI.

Methods: Females with a history of UFI were included in this cross-sectional study. They were consulted at the clinical immunology clinic at the King Abdulaziz University Hospital (KAUH). Their demographics, clinical features, total immunoglobulins, and complement test results were collected and analyzed for any relationship with UFI.

Results: One hundred and twenty-one cases of UFI with an average age of 34 ± 5.6 (range from 23 to 49 years old) were studied. Secondary infertility was predominant in 99 cases (81.8%). An overall prevalence of at least one abnormal level of total immunoglobulins or complements was found in 65 cases (55.1%). The predominant immunological abnormalities were elevated levels of immunoglobulins (hypergammaglobulinemia) in 51 cases (43.2%), high IgG in 26 cases (22%), high IgA in 14 cases (11.9%), and high IgM in 11 cases (9.3%). This was followed by elevated levels of complements (hypercomplementemia) in C4 in nine cases (8.5%). A significant association was found between the high C4 group and some parameters of infertility, including primary infertility (p = 0.005), no pregnancy (p = 0.001), and no abortion (p = 0.047), in comparison to that of the normal C4 group. Moreover, a statistically significant association was found between the high IgA group and abortion in comparison to the normal IgA group (p = 0.054).

Conclusion: At least one abnormal level of total immunoglobulins or complements was detected in more than half of the UFI cases. The commonest abnormalities were hypergammaglobulinemia (IgG, IgM, IgA) and hypocomplementenemia (C4), which showed a potential association with some infertility parameters. These findings may encourage the screening of general immunological tests to explore promising new immunopathology in UFI.

## Introduction

Unexplained female infertility (UFI) is a devastating obstetrical condition that affects females who are unable to conceive, without any definitive causes found despite extensive investigations and interventions [1,2]. The approach to UFI is continuously being updated, as the latest evidence describes different potential etiologies with clinical links, including immunological factors [2].

Immunological responses of the uterine mucosa to developing embryos are well regulated, and a successful pregnancy requires proper immune system adaptation for the fetus and placenta [2,3]. Approximately 20% of couples of reproductive age are affected by immune-mediated infertility, making it a significant health concern [3].

Immunoglobulins are vital for any immunologic evaluation to reflect the function of humoral immunity [4]. A few studies have shown that a successful pregnancy is associated with increased total IgG production in the first trimester, followed by decreased total immunoglobulin concentrations in the second and third trimesters, which results from the immunomodulation of a healthy pregnancy [5,6].

The complement system consists of a series of proteolytic enzymes and regulatory proteins that play a positive role in various pregnancy stages, such as implantation, fetal development, and labor [7]. However, an imbalance in the complement system has been detected in pregnancy complications, and this can induce unfavorable effects on both the pregnant mother and her fetus [7,8].

Although most international reproductive and obstetric societies agree that successful conception is influenced by a healthy immune system, routine immunological investigations to explore female infertility is not recommended [9]. Nevertheless, several societies recommend some immunological testing (mainly autoantibodies) for patients with recurrent pregnancy loss [10]. Recently, some societies have suggested some immunological testing for recurrent implantation failure, but with limited evidence or for clinical research purposes [11,12]. To date, there are no recommendations about testing for total immunoglobulins and complements in most reproductive societies in UFI.

However, in the face of underestimated abnormalities, general immunological laboratory investigations are seldom conducted in infertility centers in the increasing number of cases with UFI. Therefore, this research was conducted to determine the prevalence of any possible abnormalities in the levels of total immunoglobulins and complements as biomarkers in patients with UFI in the Kingdom of Saudi Arabia.

## Materials and methods

This project was a retrospective cross-sectional clinical study. It was carried out on patients with UFI who attended the clinical immunology clinic at the King Abdulaziz University Hospital (KAUH) over a period of four months, from May to August 2022. The KAUH is a tertiary referral center and a large teaching center with 800 beds, located in Jeddah City in the western zone of the Kingdom of Saudi Arabia.

This study was approved by the Unit of the Biomedical Ethics Research Committee at the KAUH, with reference number 331-22. Participants were enlightened about the purposes and procedures of the study, and participation was voluntary and without any offered incentives. Verbal consent was acquired from all participants before any collection of research data.

The inclusion criteria were specified for all participating females with unexplained infertility aged 18-50 years old who were consulted by different infertility specialists to identify any potential immunological etiologies. Other possible common causes of infertility (anatomical, genetic, and male partner factors) were excluded. Based on the World Health Organization (WHO), primary infertility occurs when a woman has never achieved a pregnancy, and secondary infertility is when at least one prior pregnancy has been achieved [13].

The criteria for exclusion were any females complaining of infertility with known common etiologies other than disturbed immunological tests, those with deficient immunoglobulins or complement laboratory results, and those who missed follow-ups.

The patients’ demographic, clinical, and laboratory information were recorded from the electronic files of medical records. Data recording was performed with Google spreadsheets for documenting the patients’ demographic data and clinical details, which included type of infertility, pregnancy number, living children, preterm labor, abortions, stillbirths, and assisted reproductive techniques, including in-vitro fertilization.

Thereafter, the laboratory results of five basic immunological tests on the serum of the included patients that were taken before attempting pregnancy were collected. These were total immunoglobulin M (IgM), immunoglobulin G (IgG), immunoglobulin A (IgA), immunoglobulin E (IgE), complement 3 (C3), and complement 4 (C4). The results of the immunological tests were obtained from the immunology laboratory at the laboratories of the KAUH.

A descriptive statistical analysis was performed for the included cases. Frequency number and percentage were extrapolated for categorical factors. The means with standard deviation were computed for the continuous variables. Then, the associations between the different collected variables were measured by the chi-square test. All p-values of < 0.05 were accounted as statistically significant. The Statistical Product and Service Solutions (SPSS, version 23) (IBM SPSS Statistics for Windows, Armonk, NY) software was utilized for all data evaluations.

This study was approved by the Research Committee of the Unit of Biomedical Ethics at the KAUH, with reference number 331-22. All participants were educated about the aims and methods of the study. The participation process was voluntary and without any offered incentives. From each participant, verbal consent was obtained before any data collection.

## Results

A total of 136 cases with UFI, referred from different specialists in infertility across Saudi Arabia, were enrolled in the clinical immunology clinic at the KAUH. Of these, 15 cases were excluded: nine were lost to follow-up and six cases for incomplete laboratory data results. In total, 121 female cases participated. The ages of participants ranged from 18 to 49 (mean age of 33.9 ± SD 5.6) years old.

The nationality data of the studied patients were 103 cases (85.1%) of Saudi citizens and 18 cases (14.9%) of non-Saudi residents. Regarding the city of cases’ residence, 70 (57.9%) were from Jeddah, 10 (8.2%) from Makkah, 10 (8.2%) from Taif, and 31 (25.6%) from other cities of Saudi Arabia (Table 1).

**Table 1:** Sociodemographic characteristics and infertility background. ICSI: intracytoplasmic sperm injection; IUI: intrauterine insemination

Parameter	Mean	SD	Minimum	Maximum	Subgroups	n	%
Age	33.95	5.57	23.00	49.00	<35	68	56.2
					>35	53	43.8
Marital status duration					≤10 years	71	58.7
					> 10 years	50	41.3
Nationality					Saudi	103	85.1
					Non-Saudi	18	14.9
City					Jeddah	70	57.9
					Other City	51	42.1
Infertility type					Primary infertility	22	18.2
					Secondary infertility	99	81.8
No. of living children	0.79	1.20	0.00	5.00	No child	74	61.2
					At least one living children	47	38.8
No. of preterm labors	0.09	0.39	0.00	3.00	No preterm labor	113	93.4
					At least one preterm	8	6.6
No. of pregnancies	3.40	3.25	0.00	14.00	No pregnancy	23	19.0
					At least one pregnancy	98	81.0
No. of abortions	2.44	2.75	0.00	14.00	No abortion	34	28.1
					At least one abortion	87	71.9
No. of stillbirth	0.11	0.40	0.00	3.00	No stillbirth	111	91.7
					At least one stillbirth	10	8.3
Intracytoplasmic sperm injection (ICSI)	1.51	1.87	0.00	10.00	No ICSI	54	44.6
					Did one ICSI or more	67	55.4
Intrauterine insemination (IUI)	0.37	0.90	0.00	4.00	No IUI	98	81.0
					Did one IUI or more	23	19.0

The background data on infertility showed that secondary infertility was predominant and had been diagnosed in 99 cases (81.8%). There were 74 cases (61.2%) who had no living children, and 47 (38.8%) who had at least one living child. At least one abortion was a prominent feature in 87 cases (71.9%), while 34 cases (28.1%) had no abortions. Regarding IVF procedures, 67 cases (55.4%) had received at least one intracytoplasmic semen injection (ICSI), and 23 cases (19%) had received at least one intrauterine insemination (IUI) (Table 1).

At least one abnormal level of any of the five biomarkers of total immunoglobulins or complements was found in 65 (55.1%) cases. Elevated immunoglobulin levels (hypergammaglobulinemia) were the commonest abnormal immunological marker, including high IgG in 26 patients (22%), followed by high IgA in 14 patients (11.9%), and high IgM in 11 patients (9.3%) (Table 2). The next most common immunological abnormality marker was elevated levels of complements (hypercomplementemia) in 10 cases (9.4%), mainly high C4 in nine of these 10 cases (8.5%) (Table 2). However, abnormally low levels of immunological markers were rare in the studied group, including low C4 in two cases (1.7%), low IgG in one case (0.8%), and low IgM in one case (0.8%).

**Table 2:** Auto-immunological antibody laboratory tests according to the prevalence. IgA: immunoglobulin A; IgG: immunoglobulin G; IgM: immunoglobulin M

	Normal		High
	N	%	N
Total IgA	104	88.1%	14
Total IgG	92	78.0%	26
Total IgM	107	90.7%	11
Hypergammaglobulinemia	84	69.4%	37
Complement C3	105	99.1%	1

A statistically significant association was detected between the high C4 group and some parameters of infertility, including primary infertility (p = 0.005), no pregnancy (p = 0.001), and no abortion (p = 0.047), more so than in the normal group (Tables 3, 4). Moreover, the high IgA group was significantly more associated with a history of at least one abortion than the normal group (p = 0.054) (Table 4).

**Table 3:** Immunoglobulin and complement correlation with sociodemographic characteristics and infertility type. * P-value significant at alpha = 0.05 IgA: immunoglobulin A; IgG: immunoglobulin G; IgM: immunoglobulin M

		Age			Marital Status Duration			Infertility type		
		<=35	>35	p-value	< = 10 years	> 10 years	p-value	Primary infertility	Secondary infertility	p-value
Total IGA	Normal	62	42	0.234	64	40	0.182	20	84	0.241
		59.6%	40.4%		61.5%	38.5%		19.2%	80.8%	
	High	6	8		6	8		1	13	
		42.9%	57.1%		42.9%	57.1%		7.1%	92.9%	
Total IGG	Normal	54	38	0.659	56	36	0.520	14	78	0.139
		58.7%	41.3%		60.9%	39.1%		15.2%	84.8%	
	High	14	12		14	12		7	19	
		53.8%	46.2%		53.8%	46.2%		26.9%	73.1%	
Total IGM	Normal	61	46	0.758	64	43	0.756	18	89	0.306
		57.0%	43.0%		59.8%	40.2%		16.8%	83.2%	
	High	7	4		6	5		3	8	
		63.6%	36.4%		54.5%	45.5%		27.3%	72.7%	
Complement C3	Normal	60	45	-	61	44	-	17	88	-
		57.1%	42.9%		58.1%	41.9%		16.2%	83.8%	
	High	0	1		0	1		0	1	
		0.0%	100.0%		0.0%	100.0%		0.0%	100.0%	
Complement C4	Normal	55	42	0.999	55	42	0.730	12	85	0.005
		56.7%	43.3%		56.7%	43.3%		12.4%	87.6%	
	High	5	4		6	3		5	4	
		55.6%	44.4%		66.7%	33.3%		55.6%	44.4%	

**Table 4:** Immunoglobulin and complement correlation with sociodemographic characteristics and infertility background. * P-value significant at alpha = 0.05 IgA: immunoglobulin A; IgG: immunoglobulin G; IgM: immunoglobulin M; IUI: intrauterine insemination; ICSI: intracytoplasmic sperm injection

		No. of Pregnancies			No. of Abortions			No. of Stillbirth			ICSI			IUI		
		No pregnancy	At least one pregnancy	p-value	No abortion	At least one abortion	p-value	No stillbirth	At least one stillbirth	p-value	No ICSI	Did one ICSI or more	p-value	No IUI	Did one IUI or more	p-value
IGA	Normal	21	83	0.239	32	72	0.054	96	8	0.942	48	56	0.213	82	22	0.214
		20.2%	79.8%		30.8%	69.2%		92.3%	7.7%		46.2%	53.8%		78.8%	21.2%	
	High	1	13		1	13		13	1		4	10		13	1	
		7.1%	92.9%		7.1%	92.9%		92.9%	7.1%		28.6%	71.4%		92.9%	7.1%	
IGG	Normal	15	77	0.220	24	68	0.392	85	7	0.989	42	50	0.514	73	19	0.549
		16.3%	83.7%		26.1%	73.9%		92.4%	7.6%		45.7%	54.3%		79.3%	20.7%	
	High	7	19		9	17		24	2		10	16		22	4	
		26.9%	73.1%		34.6%	65.4%		92.3%	7.7%		38.5%	61.5%		84.6%	15.4%	
IGM	Normal	19	88	0.440	29	78	0.515	99	8	0.848	47	60	0.922	85	22	0.360
		17.8%	82.2%		27.1%	72.9%		92.5%	7.5%		43.9%	56.1%		79.4%	20.6%	
	High	3	8		4	7		10	1		5	6		10	1	
		27.3%	72.7%		36.4%	63.6%		90.9%	9.1%		45.5%	54.5%		90.9%	9.1%	
C3	Normal	18	87	0.650	29	76	0.999	98	7	0.075	48	57	0.999	84	21	0.999
		17.1%	82.9%		27.6%	72.4%		93.3%	6.7%		45.7%	54.3%		80.0%	20.0%	
	High	0	1		0	1		0	1		0	1		1	0	
		0.0%	100.0%		0.0%	100.0%		0.0%	100.0%		0.0%	100.0%		100.0%	0.0%	
C4	Normal	13	84	0.001	24	73	0.047	90	7	0.672	46	51	0.146	78	19	0.850
		13.4%	86.6%		24.7%	75.3%		92.8%	7.2%		47.4%	52.6%		80.4%	19.6%	
	High	5	4		5	4		8	1		2	7		7	2	
		55.6%	44.4%		55.6%	44.4%		88.9%	11.1%		22.2%	77.8%		77.8%	22.2%	

In a subgroup analysis based on the age of the patients (if less than or equal to 35 years old versus older than 35 years old), there were some statistically significant associations. The high IgG group was significantly more associated with a history of no abortion than the normal group (p = 0.2). Moreover, the high C4 group was significantly associated with primary infertility (p = 0.006), no pregnancy (p = 0.006), and no abortion (p = 0.031) more than the normal group. In addition, the high IgG group was nearly significantly associated with primary infertility (p = 0.054) and no pregnancy (p = 0.056), more so than the normal group.

## Discussion

UFI, which is mainly associated with repeated abortions or implantation failures, represents an extremely challenging and distressing topic in the field of reproductive medicine. Moreover, it places a significant financial and psychological burden on the involved couples. Recent publications have advocated that an overactive immune system, such as an autoimmune disorder in some women, may increase the incidence of failed pregnancies or recurrent abortion risk [14]. This advocates a potentially greater chance of success through the evaluation of the immune system and the application of individualized immune-based treatments.

In this study, five different basic immunological laboratory biomarkers were explored in females with UFI. Interestingly, over half of the studied group had at least one abnormal test result for any of the five biomarkers of immunoglobulins or complements. A recent study measured the same five biomarkers, but during the first trimester [15]. To the best of our knowledge, this study is the first published research that evaluated these five biomarkers of immunoglobulin and complements together before pregnancy.

In the current study, participants' medical conditions are unknown. It was already known that preexisting autoimmune conditions can result in pregnancy loss [4,16]. The impacts of hypergammaglobulinemia on infertility, IVF success, and pregnancy are not yet clearly defined, but if these immunoglobulins coexist with autoantibodies, they may impair fertility [14,17].

This study revealed a potential association between high IgG and a history of (primary infertility, no pregnancy, and no abortion in younger age groups) and a near association between high IgA and a history of abortion. Preconception hypergammaglobulinemia was suggested as a risk factor for low pregnancy rates with IVF [18]. Contrary to another study, there was no relationship found between preconception immunoglobulins and recurrent abortions [19].

As expected, in this study, low levels of immunoglobulins (hypogammaglobulinemia) were found to be rare, with only one case of low IgG and one case of low IgM. Hypogammaglobulinemia is an uncommon clinical finding associated with some rare immunodeficiency disorders [4,16]. Reduced levels of IgG in the first trimester have been linked to recurrent abortions [20].

The second most predominant immunological abnormality found was high levels of complements, mainly high C4, in 9.4% of the studied group. Hypercomplementemia is seen in many inflammatory disorders as acute phase reactants [4,16]. Interestingly, the group studied in this investigation showed a relationship between preconception high C4 and a history of primary infertility, no pregnancy, and no abortion. There are few studies that have linked preconception hypercomplementemia and recurrent abortions and suggest that it may predict subsequent abortion [19].

In this studied group, hypocomplementemia was rare; low C4 was only detected in two cases (1.7%), while no participant had low C3, which is less than what has been reported in the literature. There are many studies that document preconception hypocomplementemia, more with C4 than C3, with recurrent abortions at somewhat higher rates (6-10%) and more if there are associated autoantibodies [21-24]. Hypocomplementemia is seen in immune complex diseases, which indicate consumption and disease activity or, rarely, a genetic deficiency [4,16].

This research project had a few limitations, such as the use of convenient sampling from a specific clinical immunology clinic, a few deficient patient data points, and a small sample size of cases. Hence, interpreting these immunological investigations in cases with UFI requires further large-scale, highly standard-controlled research projects in the future.

The detection of an abnormality in any of the general immunological investigations may help in the establishment of a guideline as to when and in which backgrounds of infertility to order and consider these immunological biomarkers. This might shift the perspective of experts in the field of infertility to establish a proper clinical link between the immune system and the potential causes of UFI.

## Conclusions

In conclusion, this study focused on the prevalence of five general immune biomarkers in a convenient sample of patients with UFI. Abnormal levels of at least one immunoglobulin or complement were a common finding in more than half of these patients. Among these, high immunoglobulins (IgG, IgA, IgM) and high C4 were the predominant immunological abnormalities. A potential relationship between high IgG, IgA, and C4 and lower pregnancy rates was noted. Identifying abnormal general immune responses of the mother to her fetus may advance the clinical investigational approach of UFI. Further large and randomized controlled trials for a promising clinical application of these general immunological evaluations in UFI are necessary.
